# Facile Synthesis of P-Doped ZnIn_2_S_4_ with Enhanced Visible-Light-Driven Photocatalytic Hydrogen Production

**DOI:** 10.3390/molecules28114520

**Published:** 2023-06-02

**Authors:** Xiangrui Feng, Hongji Chen, Hongfei Yin, Chunyu Yuan, Huijun Lv, Qian Fei, Yujin Zhang, Qiuyu Zhao, Mengmeng Zheng, Yongzheng Zhang

**Affiliations:** School of Physics and Physical Engineering, Qufu Normal University, Qufu 273165, Chinayinhf@qfnu.edu.cn (H.Y.); qfzhmm@163.com (M.Z.)

**Keywords:** ZnIn_2_S_4_, phosphorus doping, photocatalytic, hydrogen generation

## Abstract

ZnIn_2_S_4_ (ZIS) is widely used in the field of photocatalytic hydrogen production due to its unique photoelectric properties. Nonetheless, the photocatalytic performance of ZIS usually faces problems of poor conductivity and rapid recombination of charge carriers. Heteroatom doping is often regarded as one of the effective strategies for improving the catalytic activity of photocatalysts. Herein, phosphorus (P)-doped ZIS was prepared by hydrothermal method, whose photocatalytic hydrogen production performance and energy band structure were fully studied. The band gap of P-doped ZIS is about 2.51 eV, which is slightly smaller than that of pure ZIS. Moreover, due to the upward shift of its energy band, the reduction ability of P-doped ZIS is enhanced, and P-doped ZIS also exhibits stronger catalytic activity than pure ZIS. The optimized P-doped ZIS exhibits a hydrogen production rate of 1566.6 μmol g^−1^ h^−1^, which is 3.8 times that of the pristine ZIS (411.1 μmol g^−1^ h^−1^). This work provides a broad platform for the design and synthesis of phosphorus-doped sulfide-based photocatalysts for hydrogen evolution.

## 1. Introduction

The excessive use of fossil fuels has inevitably led to an energy crisis and severe environmental problems [1]. Therefore, it is essential to identify a source of clean and renewable energy. Hydrogen (H_2_) is deemed one of the most prospective energy sources because of its remarkable energy density and zero carbon emission from combustion [2]. Since the discovery of water splitting for hydrogen production over TiO_2_ under UV light irradiation [3], photocatalytic technology has exhibited great potential for hydrogen production [4,5,6,7]. Generally, light absorption, charge separation and transfer, as well as redox reaction on the surface, are involved with the photocatalytic process [8,9]. Therefore, the construction of photocatalytic systems with broad light absorption and enhanced charge separation is highly demanded.

Among many types of photocatalytic materials, ZnIn_2_S_4_ has displayed significant potential in water splitting owing to its suitable band structure, visible light absorption, and stable chemical properties [10,11,12,13,14,15,16]. However, the photocatalytic efficiency of ZIS is still by no means satisfactory because of serious charge recombination. Various strategies have been adopted to optimize the photocatalytic performance of the pristine ZIS, including vacancy engineering [17,18], cocatalyst loading [19,20], hetero-atom doping [21,22], and coupling with other semiconductor materials [23,24,25]. In addition, the controllable design of heterojunctions and macro-nanostructure have been effectively applied to change the surface properties and electronic structure of ZIS, thus enhancing its ability of photocatalytic hydrogen evolution [26,27]. It is reported that the cation-doped ZIS exhibited enhanced light absorption, however, the doped cations can be the recombination center of the charge carriers [28,29]. Nonmetal doping has emerged as a substitution. For example, Du et al. reported an N-doped ZIS for hydrogen production by using DMF as a solvent in the hydrothermal process, the doping of the N element promotes the separation of charge carriers and elevates the CB potential to produce more reductive photoelectrons for hydrogen evolution [21]. Xie et al. prepared an oxygen-doped ZIS by adding PVP before the hydrothermal route. The incorporation of oxygen atoms increases the lifetime of photoelectrons, leading to an enhanced photocatalytic performance [22].

However, in the current photocatalytic applications, P doping has been mainly applied in TiO_2_ and g-C_3_N_4_ materials. There are few research reports on P-doped ZIS, and the actual mechanism of P doping on band structure and charge transfer is also limited. In this work, P-doped ZIS was synthesized by a straightforward hydrothermal method using NaH_2_PO_2_ as the P source, which showed outstanding photocatalytic activity toward hydrogen evolution. The regulation of band structure and photocatalytic performance are facilely realized by changing the amount of NaH_2_PO_2_. Through the systematical characterizations of light absorption and the transport behavior of charge carriers, the reason for the enhanced photocatalytic performance is revealed. P-doped ZIS has a wider light absorption range and a narrower band gap than pure ZIS, which improves the utilization of visible light. In addition, P-doped ZIS achieves faster charge migration due to its low resistance. The upward shift of the band structure also gives it a higher reduction ability. This work provides a broad platform for the design and synthesis of phosphorus-doped sulfide-based photocatalysts for hydrogen evolution.

## 2. Results and Discussion

### 2.1. Morphology and Structure of Photocatalysts

**Figure 1:**
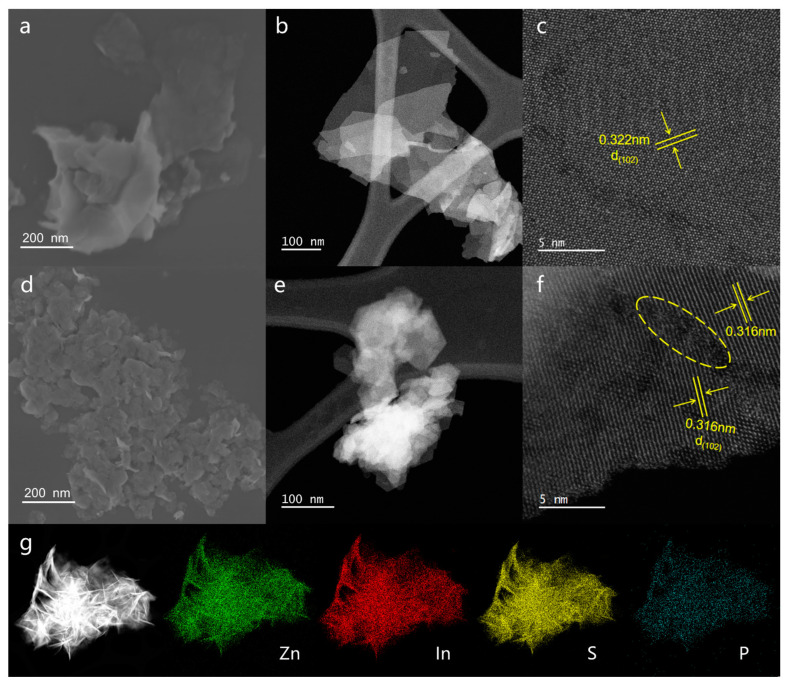
Morphology and microstructure analysis of the pure ZIS and P-ZIS-1.0: SEM images of (**a**) pure ZIS and (**d**) P-ZIS-1.0; TEM and HRTEM images of (**b**,**c**) pure ZIS and (**e**,**f**) P-ZIS-1.0; (**g**) EDS elemental mapping of P-ZIS-1.0.

The morphology and microstructure of pure ZIS and P-doped ZIS were analyzed by SEM and TEM. Figure 1a,b displays the SEM and TEM images of pure ZIS, whose morphology is a two-dimensional nanosheet structure. The uniform dispersion of Zn, In, and S in Figure S1 demonstrates the successful synthesis of ZIS nanosheets. The lattice spacing of 0.322 nm can be indexed into the (102) crystal plane of hexagonal ZIS (Figure 1c). After phosphorus doping, the obtained P-ZIS-1.0 exhibits a smaller and curlier nanosheet structure, as shown in Figure 1d,e. The smaller nanosheet structure can expose more active sites at the edge, which is beneficial to the photocatalytic activity of P-ZIS. Notably, the HRTEM image of P-ZIS-1.0 shows discontinuous lattice stripes (dashed circles in Figure 1f), indicating significant structural distortion in the P-doped ZIS nanosheets. Meanwhile, the lattice spacing corresponding to the (102) crystal plane is slightly smaller than the pristine ZIS, which may be attributed to the introduction of internal stress in the lattice of P-doped ZIS [30]. Furthermore, the energy-dispersive spectrum (EDS) element mapping image of the selected region (Figure 1g) displays the uniform distribution of P, Zn, In, and S elements in P-doped ZIS samples, further confirming the successful preparation of P-doped ZIS.

**Figure 2:**
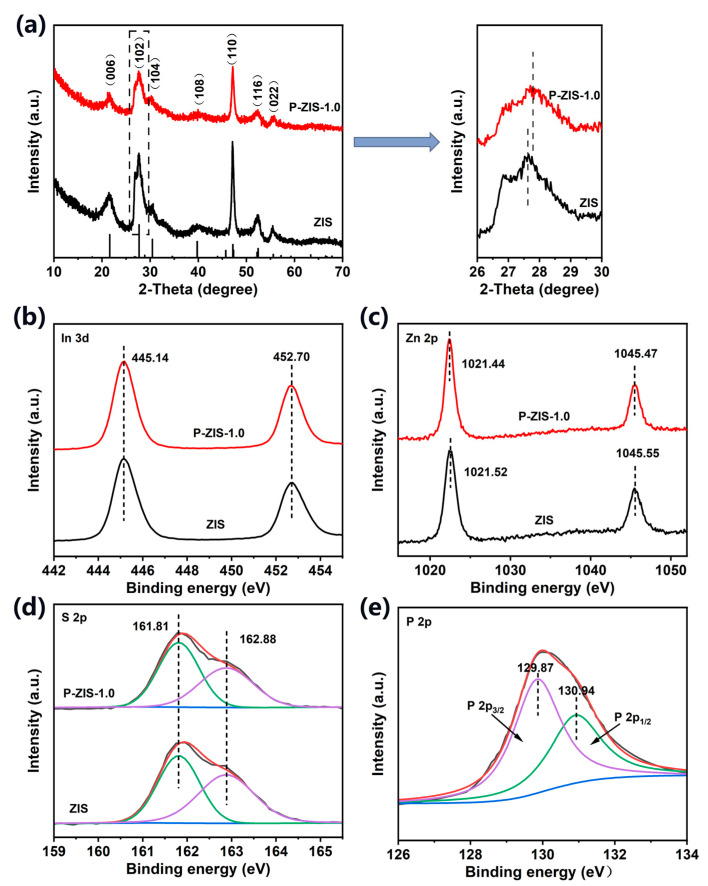
(**a**) XRD patterns of the pristine ZIS and P-ZIS-1.0; XPS survey spectra of ZIS and P-ZIS-1.0: (**b**) In 3d; (**c**) Zn 2p; (**d**) S 2p; and (**e**) P 2p.

The XRD patterns of the pristine ZIS and P-ZIS-1.0 are displayed in Figure 2a. It can be clearly seen that the diffraction peaks of pristine ZIS are in keeping with the hexagonal phase of ZnIn_2_S_4_ (JCPDS card No.65-2023), and the XRD pattern of P-ZIS-1.0 is basically consistent with the pristine ZIS. After doping with P atoms, the structure of the hexagonal lattice remains unchanged, and no impurity phases are generated, proving that the doping of P atoms does not change the original lattice structure of ZIS. However, compared with pure ZIS, the relative intensity of diffraction peaks in P-ZIS-1.0 samples is decreased, indicating that P doping has an inhibitory effect on crystal growth [31]. It is worth noting that in the amplified XRD patterns, the diffraction peak corresponding to the (102) crystal plane of P-ZIS-1.0 shifts towards a higher angle as compared to the original ZIS, which is consistent with the decreased lattice spacing. This phenomenon can also be observed in the other P-doped ZIS samples, as shown in Figure S2. The more P source, the more positive shift of the characteristic diffraction peak corresponding to the (102) crystal plane.

To clarify the composition and chemical state of ZIS and P-ZIS-1.0, XPS tests were performed. Figure S3 displays the full XPS spectra of the fabricated ZIS and P-ZIS-1.0, where the signals of Zn, In, and S can be observed in the pristine ZIS. Meanwhile, the presence of the signal of P in the P-ZIS-1.0 confirms the successful doping of P atoms into the ZIS. As shown in Figure 2b, the two characteristic peaks at 445.14 and 452.70 eV belong to In 3d_5/2_ and In 3d_3/2_ of In^3+^, respectively [32]. For Zn 2p (Figure 2c), the signals at a binding energy of 1021.52 and 1045.55 eV in the pristine ZIS can be attributed to the Zn 2p_3/2_ and Zn 2p_1/2_ of Zn^2+^ [33]. Compared with the pristine ZIS, the characteristic peaks of Zn 2p in the P-ZIS-1.0 shift to lower binding energy, indicating the doping of P atoms changes the chemical environment of the Zn atom. In addition, as shown in Figure 2d, the two characteristic peaks at 161.81 and 162.88 eV belong to S 2p_3/2_ and S 2p_1/2_ of S^2−^. Compared with the pristine ZIS, a slight decrease in peak intensity can be observed in P-ZIS-1.0, indicating the S atom has been partially replaced [34]. In terms of P 2p, the characteristic signal can be deconvoluted into two peaks at 129.87 and 130.94 eV, belonging to P 2p_3/2_ and P 2p_1/2_, respectively, which can be inferred as P-Zn or P-In bonds [35,36]. In summary, it is believed that the phosphorus atoms were introduced by replacing S atoms and forming Zn-P bonds.

In addition, the specific surface areas of the fabricated ZIS and P-ZIS-1.0 were measured by N_2_ adsorption-desorption technology. The nitrogen adsorption-desorption isotherm of pure ZIS and P-ZIS-1.0 are shown in Figure S4. All the samples exhibit the type IV isotherms with a hysteresis loop at the high-relative-pressure (P/P_0_) region, suggesting the existence of a mesoporous structure. Furthermore, the high absorption in the high relative pressure range (approaching 1.0) in each isotherm also indicates the formation of large mesopores and macropores [37]. The BET surface area of P-ZIS-1.0 (71.64 m^2^ g^−1^) is larger than that of pure ZIS (54.18 m^2^ g^−1^). Since a higher specific surface area may have a better adsorption ability and provide a more active site to adsorb more reactive substances [38], this is beneficial for optimizing the photocatalytic performance of P-ZIS-1.0.

### 2.2. Photocatalytic Hydrogen Evolution Activity

**Figure 3:**
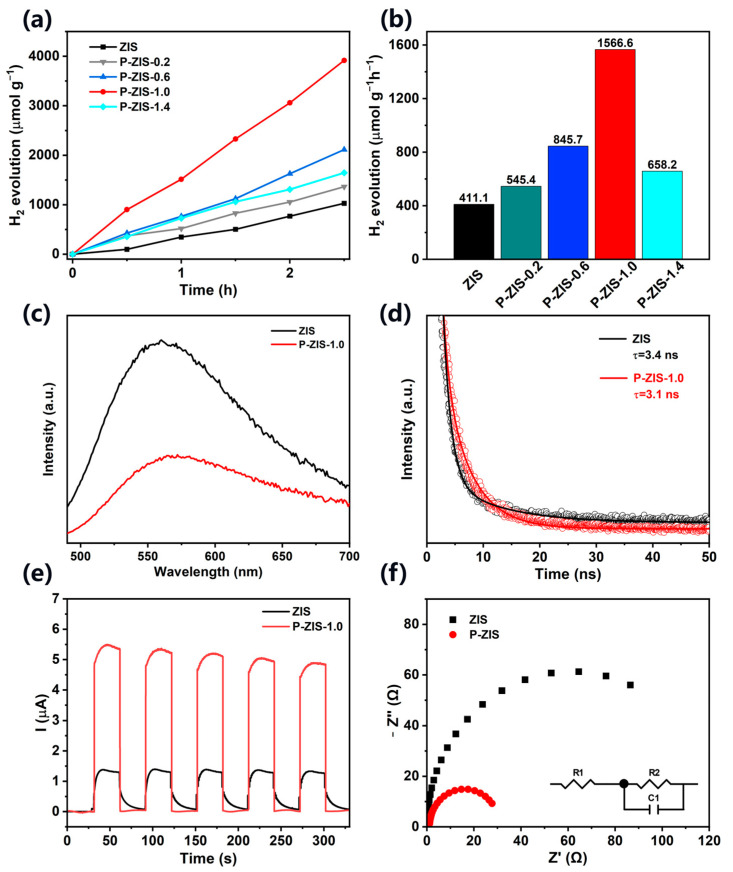
(**a**) Time-dependent diagrams of photocatalytic H_2_ evolution among pure ZIS and different P-doped ZIS; (**b**) the hydrogen production rate of pure ZIS and different P-doped ZIS; (**c**) PL spectra; (**d**) TRPL spectra; (**e**) I-t curves; and (**f**) EIS plots of the pure ZIS and P-ZIS-1.0, inset image: Equivalent circuit diagram.

The photocatalytic hydrogen evolution activity of the fabricated samples was performed under visible light illumination, as exhibited in Figure 3a,b. It can be observed that hydrogen production increased with the prolonging of irradiation time, but the pristine ZIS exhibits an unsatisfactory hydrogen generation performance, which possesses a hydrogen evolution rate of 411.1 μmol g^−1^ h^−1^. Fortunately, all the P-doped samples display enhanced photocatalytic performance. The hydrogen evolution rate over the fabricated P-ZIS-0.2, P-ZIS-0.6, P-ZIS-1.0, and P-ZIS-1.4 is 545.4, 845.7, 1566.6, and 658.2 μmol g^−1^ h^−1^, respectively. At the same time, the turnover frequency of pure ZIS and P-ZIS-1.0 is calculated (Table 1). Compared with the pristine ZIS (0.174 h^−1^), the turnover frequency of optimized P-ZIS-1.0 is 0.663 h^−1^, which is approximately 3.8 times as high as the pristine ZIS. In addition, by combining the specific surface areas of the fabricated ZIS and P-ZIS-1.0 with the hydrogen evolution, the hydrogen production rate of P-ZIS-1.0 is 21.9 μmol m^−2^ h^−1^, which is higher than that of the pristine ZIS (7.5 μmol m^−2^ h^−1^), demonstrating the enlarged specific surface areas is in favor of the photocatalytic reaction. In addition, the AQY of P-ZIS-1.0 is estimated to be 3.2% at 420 nm (Figure S5). The enhanced photocatalytic performance can be attributed to the introduction of P atoms having a positive effect on the transportation behavior of charge carriers. However, the hydrogen evolution performance is not just increased with the increase of P-doping. The excess doping of P atoms into the ZIS framework may generate unfavorable lattice distortion and form an undesirable charge recombination center [39].

The cyclic stability of a photocatalyst is another important factor affecting its practical application. Therefore, it is important to evaluate the stability of a photocatalyst during photocatalysis. In order to check the stability of the fabricated P-ZIS-1.0, the catalyst was centrifuged for recovery and washed several times with deionized water and absolute ethanol, then dried at 60 °C overnight for cyclic photocatalytic hydrogen evolution testing. Compared with the first recycle run, there is just an attenuation of 9.5% in the fifth recycle run, implying the fabricated P-ZIS-1.0 is stable (Figure S6a). The reused P-ZIS-1.0 was collected and characterized by XRD, as shown in Figure S6b. There is no obvious change can be observed between the fresh and reused P-ZIS-1.0, confirming the structural stability of the as-prepared P-ZIS-1.0. More importantly, the P-doped ZnIn_2_S_4_ shows superior photocatalytic hydrogen production performance as compared to some other ZnIn_2_S_4_-based photocatalysts reported in the past two years (Table S1), which further indicates that P-doped ZIS has been rationally modified by doping with P for favorable photocatalytic hydrogen evolution performance.

### 2.3. Charge Transfer Dynamics and Energy Band Structures Analysis

To reveal the underlying causes of the improved photocatalytic hydrogen evolution performance over the fabricated P-doped ZIS, various characterizations were carried out. The separation and migration efficiency of the photogenerated electron-hole pairs is evaluated by steady-state PL, time-resolved photoluminescence (TRPL), and photoelectrochemical measurements. Figure 3c displays the PL spectra of the obtained ZIS and P-ZIS-1.0. In general, the recombination rate of photoexcited holes and electrons can be inferred from the PL emission intensity, the lower the PL emission intensity, the lower the recombination rate of photoexcited holes and electrons. Compared with the pure ZIS, the as-prepared P-ZIS-1.0 exhibits a lower peak intensity, indicating that P-ZIS-1.0 has a higher charge separation efficiency [40,41]. Figure 3d shows the TRPL spectra of the pristine ZIS and P-ZIS-1.0, where the lifetime of charge carriers in the original ZIS is 3.4 ns, while the lifetime of charge carriers in P-ZIS-1.0 (3.1 ns) is slightly smaller than that of ZIS. Owing to lower charge carrier lifetimes reflecting a stronger charge separation ability [42,43], the shorter lifetime of charge carriers of P-ZIS-1.0 than that of pristine ZIS implies the doping of P element enhances the separation ability of photoexcited holes and electrons in ZIS. The transient photocurrent response was performed to further investigate the charge separation. The current-time curves that have several on-off cycles under discontinuous visible light irradiation for ZIS and P-ZIS in 0.5 M Na_2_SO_4_ aqueous solution are illustrated in Figure 3e. The transient photocurrent density of the P-ZIS-1.0 is higher than that of the original ZIS, indicating a more efficient charge separation, owing to the photocurrent density and charge separation efficiency being positively correlated [44,45]. EIS is employed to analyze the transfer efficiency of the charge carriers, as exhibited in Figure 3f. Compared with the pristine ZIS, the P-ZIS-1.0 displays a smaller semicircle in the Nyquist plots, indicating that P-ZIS-1.0 has a lower charge transfer resistance and higher electron mobility [46,47]. In addition, an equivalent circuit for fitting the impedance spectra is shown in the inset pattern, where C1 is the space charge capacitor corresponding to the ability to store charges in the semiconductor/electrolyte interface bilayer, R2 is the charge transfer resistance at the same interface in Faraday response. As the frequency of the excitation source increases, the Faraday impedance approaches R2, thus a semicircle is observed in the Nyquist plot. For low frequencies, Faraday impedance can be considered as two resistors connected in series, one attributed to electron transfer at the semiconductor/electrolyte interface and the other attributed to mass transfer towards the electrode. R1 represents series resistance, including resistance related to ion conductivity in the electrolyte and external contact resistance [48,49]. In a word, PL, TRPL, transient photocurrent response, and EIS results all indicate that the introduction of P is beneficial for promoting the separation and transfer of charge carriers in ZIS, thereby promoting photocatalytic hydrogen production.

**Figure 4:**
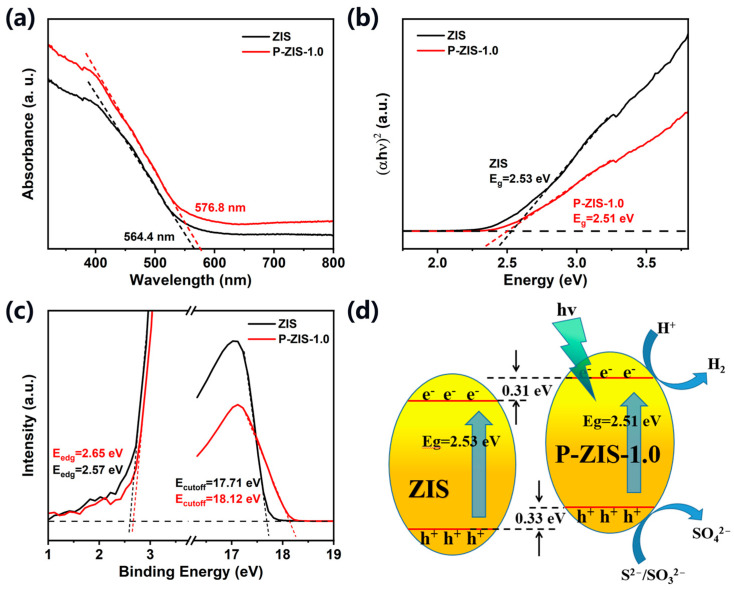
(**a**) UV-vis DRS spectra of ZIS and P-ZIS-1.0; (**b**) the band gap value curves of ZIS and P-ZIS-1.0 obtained from UV-vis DRS spectra; (**c**) UPS of ZIS and P-ZIS-1.0; (**d**) schematic diagram of the energy band structure of P-ZIS-1.0 and pure ZIS.

It is well known that element doping also has a significant impact on the energy band structure of semiconductors and thus affects their photocatalytic activity. Figure 4a shows the UV-vis DRS of the ZIS and P-ZIS-1.0. Both pure ZIS and P-ZIS-1.0 show good light absorption ability in the visible light region. Meanwhile, the absorption edge of the P-ZIS-1.0 is a little broader than that of the pristine ZIS, corresponding to a smaller bandgap energy. The bandgap of a semiconductor can be estimated by using the formula described as follow: *αhv* = *A*(*hv* − *E_g_*)^1/2^, where *hv*, *α*, *A*, and *E_g_* correspond to solar light energy, absorption index, constant value, and bandgap energy of semiconductor, respectively. As shown in Figure 4b, the bandgap of P-ZIS-1.0 is figured out to be 2.51 eV, which is slightly smaller than that of pristine ZIS (2.53 eV) and other P-doped ZIS (Figure S7). This also indicates that P-ZIS-1.0 has excellent photocatalytic performance, owing to the broader light absorption.

To obtain the band edge position of the fabricated ZIS and P-ZIS-1.0, ultraviolet photoelectron spectroscopy (UPS) tests were further carried out, as shown in Figure 4c. The work function (ϕ) of pure ZIS is calculated to be 3.51 eV, and the valence band edge (VB) is +1.54 V (vs. NHE). In the same way, the ϕ and E_VB_ of P-ZIS-1.0 is 3.1 and 1.31 V (vs. NHE), respectively, the details of the calculation process are provided in the supporting information. Based on the bandgap of the pristine ZIS and P-ZIS-1.0, the conduction band (CB) of the ZIS and P-ZIS-1.0 is −0.89 and −1.20 eV, respectively. The mild reduction ability of the prepared samples ensures that it is thermodynamically feasible to reduce H^+^ as a photocatalyst. Moreover, compared to ZIS, the P-ZIS-1.0 has a stronger reduction ability and can better perform H^+^ reduction [50,51]. In summary, P-doped ZIS has a smaller band gap, which is more conducive to electron excitation. It has a higher conduction band position and stronger reduction ability, making it more suitable for photocatalytic hydrogen production than pure ZIS.

The energy level diagram of pure ZIS and P-ZIS-1.0 plotted based on band gaps and energy levels is shown in Figure 4d. According to the above-mentioned characterizations and analysis, the enhanced photocatalytic performance of the P-ZIS-1.0, as compared to the pristine ZIS can be attributed to the promotion of charge separation and effective inhibition of electron-hole recombination during the photocatalytic process. P-ZIS-1.0 has better carrier transfer ability and a smaller bandgap. Moreover, the upward movement of the CB edge of P-ZIS-1.0 indicates that it has a stronger reduction ability and can better carry out reduction reactions, enabling the reaction to proceed forward [52,53]. The comprehensive synergistic effect of the above advantages contributes to the improvement of the photocatalytic hydrogen production efficiency over P-ZIS-1.0.

## 3. Materials and Methods

### 3.1. Synthesis of P-Doped ZIS and Pure ZIS

P-doped ZIS and pure ZIS nanosheets were synthesized by the one-step hydrothermal method. Generally, 0.5 mmol of zinc acetate (Zn(CHCOO)_2_, 99.9%, Aladdin, Shanghai, China), 1.0 mmol of indium trichloride (InCl_3_, 99.9%, Aladdin Shanghai, China), and 2.0 mmol of thioacetamide (CH_3_CSNH_2_, 98.0%, Aladdin Shanghai, China) were dissolved in 60 mL of deionized water and stirred for 30 min. Then, the apparent solutions were transferred into a 100 mL autoclave and heated at 180 °C for 12 h. The obtained sample was washed with distilled water and ethanol three times and dried at 60 °C overnight, denoted as ZIS. The preparation process of P-doped ZIS was the same as the above-mentioned, except for adding a certain amount of sodium monophosphate (NaH_2_PO_2_, 99%, Innochem, Beijing, China) into the precursor before the hydrothermal process. The samples fabricated by adding 0.2, 0.6, 1.0, and 1.4 mmol NaH_2_PO_2_ were labeled as P-ZIS-x (x = 0.2, 0.6, 1.0, and 1.4), where x represented the molar mass of the added NaH_2_PO_2_.

### 3.2. Characterization

The X-ray diffraction (XRD) patterns of the fabricated ZIS and P-doped ZIS were obtained by the Rigaku D/Max-cA (Tokyo, Japan) diffractometer at Cu-Kα radiation. The morphology, microstructure, and element distribution of the as-prepared ZIS and P-doped ZIS-1.0 were observed on the field emission scanning electron microscopy (FE-SEM, Carl Zeiss Ultra 55, Oberkochen, Germany) and transmission electron microscopy (TEM, JEOL-ARM200F, Tokyo, Japan). The composition and ultraviolet photoelectron spectroscopy (UPS) of the fabricated samples were analyzed by X-ray photoelectron spectroscopy measurements (XPS, AXIS SUPRA+, SHIMADZU, Manchester, UK). The specific surface areas of the pristine ZIS and P-ZIS-1.0 were determined by N_2_ adsorption-desorption measurements (Osaka, BEL. JAPAN. INC, at 77.0 K) with the Brunauer–Emmett–Teller (BET) methods. UV-vis diffuse reflectance spectra (UV-vis DRS) of the fabricated samples were obtained by UV-vis spectrophotometer (Lambda 1050, PerkinElmer, Waltham, MA, USA). The photoluminescence (PL) emission spectra and the time-resolved fluorescence decay spectra (TRPL) were measured by an ambient temperature fluorescence spectrophotometer (Edinburgh, FLS1000, Scotland, UK).

### 3.3. Photocatalytic Activity

In the homogeneous solution of Na_2_S/Na_2_SO_3_ mixture with the concentration of 0.25 M/0.35 M as hole scavengers, 25 mg of photocatalysts were dispersed in a 60 mL aqueous solution. Before illumination, the 250 mL reaction cell was installed in CEL-PAEM-D8Pro equipment (Beijing China Education Au-light Co., Ltd., Beijing, China). The wavelength range of illumination was 420 nm < *λ* < 760 nm, which originated from a 300 W Xe lamp. To guarantee the favorable airtightness of the photocatalytic reaction system, the photocatalytic diffusion system was vacuumed for 20 min with slow stirring of quartz glass reaction cell ahead of schedule. The photocatalytic quartz glass reaction cell was kept stirring at a fixed rotation speed at 300 rpm for the as-measured samples, and the cooling water circulation system with a tube-in-tube condenser was employed to control the reaction temperature at 6 °C. Molecular 5 A column and TCD were configured on Shimadzu GC-2014 chromatography to analyze the generated hydrogen in real-time every half hour.

This is an equation for turnover frequency (TOF) of the catalysts:

(1) $$TON=\frac{moles\;of\;evoluted\;H_{2}}{moles\;of\;photocatalyst}\;TOF=\frac{TON}{reaction\;time\;(h)}$$

The light intensity (*P*) was determined by an optical power meter to calculate the apparent quantum yields (AQY) at 420 nm wavelength, as shown in the following equation:

(2) $$AQY=\frac{2\times number\;of\;evolved\;H_{2}\;molecules}{the\;number\;of\;incident\;photons}\times 100\%=\frac{2MN_{A}hc}{SPT\lambda }\times 100\%$$

where *M* is the amount of produced H_2_ molecule (mol), *N_A_* is the Avogadro constant (6.022 × 10^23^ mol^−1^), *h* is the Planck constant (6.626 × 10^−34^ J s^−1^), *c* is the speed of light (3 × 10^8^ m s^−1^), *λ* is the wavelength of the incident light (*λ* = 4.2 × 10^−7^ m), *P* is the light intensity (100 mW cm^−2^), *S* is the surface area of the irradiated photoreactor (19.64 cm^2^), and *T* is the photocatalytic hydrogen evolution time (9000 s).

### 3.4. Photoelectrochemical Measurements

The transient photocurrent response and electrochemical impedance spectroscopy (EIS) were carried out in a standard three-electrode quartz cell on a CHI-760E electrochemical workstation. This system is equipped with a 300 W Xe lamp with a 420 nm cut-off filter as the visible light source. A 0.5 M Na_2_SO_4_ aqueous solution was utilized as the electrolyte. Ag/AgCl is used as the reference electrode, and a Pt plate electrode is used as the counter electrode. The working electrode was prepared as follows: 4 mg of photocatalyst powder was dispersed into a mixed solution containing 750 μL of deionized water, 250 μL of absolute ethanol, and 10 μL of 5% Nafion. The mixture was subjected to ultrasonic treatment for 60 min to form a homogeneous dispersion. Next, 200 μL of the above mixture was loaded onto the fluorine-doped tin oxide glass (1 × 3 cm) with a coating area of 1 cm^2^.

## 4. Conclusions

To sum up, P-doped ZIS nanosheets were successfully prepared via a facile one-step hydrothermal method, which exhibits superior photocatalytic hydrogen evolution performance. The doping of phosphorus atoms improves the utilization of visible light, changes the energy band structure of ZIS, promotes charge transfer, and prohibits the recombination of electron-hole pairs. The upward shift of the band structure enhances the reduction ability of ZIS, which is conducive to photocatalytic reduction reactions and improves the activity of photocatalytic hydrogen production. Compared with the original ZIS, the hydrogen evolution rate of the as-prepared P-ZIS-1.0 (1566.6 μmol g^−1^ h^−1^) is 3.8 times that of undoped ZIS (411.1 μmol g^−1^ h^−1^). More importantly, P-doped ZnIn_2_S_4_ exhibits excellent performance compared to other modified catalysts based on ZnIn_2_S_4_. This work provides a broad platform for the design and synthesis of phosphorus-doped sulfide-based photocatalysts for hydrogen evolution.

## Figures and Tables

**Table 1:** Comparison of TOF, hydrogen generation rate, and BET surface area between ZIS and P-ZIS-1.0.

Samples	TOF (h^−1^)	H_2_ Generation (μmol g^−1^ h^−1^)	BET (m^2^ g^−1^)	H_2_ Generation/BET (μmol m^2^ h^−1^)
ZIS	0.174	411.1	54.18	7.5
P-ZIS-1.0	0.663	1566.6	71.64	21.9

## Data Availability

The data presented in this study are available on request from the author.

## Supplementary Materials

The following supporting information can be downloaded at: https://www.mdpi.com/article/10.3390/molecules28114520/s1, Figure S1: Energy disperse spectrum elemental mapping of the pure ZnIn_2_S_4_.; Figure S2: XRD patterns of the as-prepared photocatalysts.; Figure S3: High-resolution XPS survey spectra of ZIS and P-ZIS-1.0.; Figure S4: BET spectra of ZIS and P-ZIS-1.0.; Figure S5: AQY (%) of different samples.; Figure S6: (a) Recycling photocatalytic H_2_ generation tests over P-ZIS-1.0. (b) XRD of the fresh and used P-ZIS-1.0 samples.; Figure S7: (a) UV-vis DRS of the as-prepared P-ZIS.; (b) The band gap value curves of the as-prepared photocatalysts obtained from UV–vis DRS spectra.; Table S1: Comparison of the performance of P-ZIS-1.0 with other recently reported improved materials based on ZIS. Figure S8: Schematic illustration of the energy level calculation from UPS. References [54,55,56,57,58,59,60,61,62,63,64,65,66] are cited in the supplementary materials.

## Sample Availability

Samples of the compounds are available from the authors.

## References

[1] Fu C.F., Wu X., Yang J. (2018). Material design for photocatalytic water splitting from a theoretical perspective. Adv. Mater..

[2] Wang Q., Domen K. (2020). Particulate photocatalysts for light-driven water splitting: Mechanisms, challenges, and design strategies. Chem. Rev..

[3] Fujishima A., Honda K. (1972). Electrochemical photolysis of water at a semiconductor electrode. Nature.

[4] Tao X.P., Zhao Y., Wang S.Y., Li C., Li R.G. (2022). Recent advances and perspectives for solar-driven water splitting using particulate photocatalysts. Chem. Soc. Rev..

[5] Song H., Luo S.Q., Huang H.M., Deng B.W., Ye J.H. (2022). Solar-driven hydrogen production: Recent advances, challenges, and future perspectives. ACS Energy Lett..

[6] Zhang Y.M., Zhao J.H., Wang H., Xiao B., Zhang W., Zhao X.B., Lv T.P., Thangamuthu M., Zhang J., Guo Y. (2022). Single-atom Cu anchored catalysts for photocatalytic renewable H_2_ production with a quantum efficiency of 56%. Nat. Commun..

[7] Ruan X.W., Cui X.Q., Cui Y., Fan X.F., Li Z.Y., Xie T.F., Ba K.K., Jia G.R., Zhang H.Y., Zhang L. (2022). Favorable energy band alignment of TiO_2_ anatase/rutile heterophase homojunctions yields photocatalytic hydrogen evolution with quantum efficiency exceeding 45.6%. Adv. Energy Mater..

[8] Kumar A., Raizad P., Singh P., Saini R.V., Sain A.K., Ahmad H.-B. (2020). Perspective and status of polymeric graphitic carbon nitride based Z-scheme photocatalytic systems for sustainable photocatalytic water purification. Chem. Eng. J..

[9] Li H.J., Zhou Y., Tu W.G., Ye J.H., Zou Z.G. (2015). State-of-the-art progress in diverse heterostructured photocatalysts toward promoting photocatalytic performance. Adv. Funct. Mater..

[10] Wang J., Sun S.J., Zhou R., Li Y.Z., He Z.T., Ding H., Chen D.M., Ao W.H. (2021). A review: Synthesis, modification and photocatalytic applications of ZnIn_2_S_4_. J. Mater. Sci. Technol..

[11] Yadav G., Ahmaruzzaman M. (2022). ZnIn_2_S_4_ and ZnIn_2_S_4_ based advanced hybrid materials: Structure, morphology and applications in environment and energy. Inorg. Chem. Commun..

[12] Wang Y.J., Huang W.J., Guo S.H., Xin X., Zhang Y.Z., Guo P., Tang S.W., Li X.H. (2021). Sulfur-deficient ZnIn_2_S_4_/oxygen-deficient WO_3_ hybrids with carbon layer bridges as a novel photothermal/photocatalytic integrated system for Z-scheme overall water splitting. Adv. Energy Mater..

[13] Zhang G.P., Wu H., Chen D.Y., Li N.J., Xu Q.F., Li H., He J.H., Lu J.M. (2022). A mini-review on ZnIn_2_S_4_-based photocatalysts for energy and environmental application. Green Energy Environ..

[14] Cai Y., Luo F.X., Guo Y.J., Guo F., Shi W.L., Yang S.T. (2023). Near-infrared light driven ZnIn_2_S_4_-based photocatalysts for environmental and energy applications: Progress and perspectives. Molecules.

[15] Zheng X.L., Song Y.M., Liu Y.H., Yang Y.Q., Wu D.X., Yang Y.J., Feng S.Y., Li J., Liu W.F., Shen Y.J. (2023). ZnIn_2_S_4_-based photocatalysts for photocatalytic hydrogen evolution via water splitting. Coord. Chem. Rev..

[16] Ren Y.J., Foo J.J., Zeng D.Q., Ong W.J. (2022). ZnIn_2_S_4_-based nanostructures in artificial photosynthesis: Insights into photocatalytic reduction toward sustainable energy production. Small Struct..

[17] Meng S.G., Chen C., Gu X.M., Wu H.H., Meng Q.Q., Zhang J.F., Chen S.F., Fu X.L., Liu D., Lei W.W. (2021). Efficient photocatalytic H_2_ evolution, CO_2_ reduction and N_2_ fixation coupled with organic synthesis by cocatalyst and vacancies engineering. Appl. Catal. B Environ..

[18] Jing X.D., Lu N., Huang J.D., Zhang P., Zhang Z.Y. (2021). One-step hydrothermal synthesis of S-defect-controlled ZnIn_2_S_4_ microflowers with improved kinetics process of charge-carriers for photocatalytic H_2_ evolution. J. Energy Chem..

[19] Si S.H., Shou H.W., Mao Y.Y., Bao X.L., Zhai G.Y., Song K.P., Wang Z.Y., Wang P., Liu Y.Y., Zheng Z.K. (2022). Low-coordination single Au atoms on ultrathin ZnIn_2_S_4_ nanosheets for selective photocatalytic CO_2_ reduction towards CH_4_. Angew. Chem. Int. Ed..

[20] Shi X.W., Dai C., Wang X., Hu J.Y., Zhang J.Y., Zheng L.X., Mao L., Zheng H.J., Zhu M.S. (2022). Protruding Pt single-sites on hexagonal ZnIn_2_S_4_ to accelerate photocatalytic hydrogen evolution. Nat. Commun..

[21] Du C., Yan B., Lin Z., Yang G. (2020). Enhanced carrier separation and increased electron density in 2D heavily N-doped ZnIn_2_S_4_ for photocatalytic hydrogen production. J. Mater. Chem. A.

[22] Yang W.L., Zhang L., Xie J.F., Zhang X.D., Liu Q.H., Yao T., Wei S.Q., Zhang Q., Xie Y. (2016). Enhanced photoexcited carrier separation in oxygen-doped ZnIn_2_S_4_ nanosheets for hydrogen evolution. Angew. Chem. Int. Ed..

[23] Liang Q., Gao W., Liu C.H., Xu S., Li Z.Y. (2020). A novel 2D/1D core-shell heterostructures coupling MOF-derived iron oxides with ZnIn_2_S_4_ for enhanced photocatalytic activity. J. Hazard. Mater..

[24] Liu H., Zhang J., Ao D. (2018). Construction of heterostructured ZnIn_2_S_4_@NH_2_-MIL-125(Ti) nanocomposites for visible-light-driven H_2_ production. Appl. Catal. B Envrion..

[25] Wang L.B., Cheng B., Zhang L.Y., Yu J.G. (2021). In situ irradiated XPS investigation on S-scheme TiO_2_@ZnIn_2_S_4_ photocatalyst for efficient photocatalytic CO_2_ reduction. Small.

[26] Wang S., Guan B.Y., Lou X.W.D. (2018). Construction of ZnIn_2_S_4_-In_2_O_3_ hierarchical tubular heterostructures for efficient CO_2_ photoreduction. J. Am. Chem. Soc..

[27] Li W., Lin Z., Yang G. (2017). A 2D self-assembled MoS_2_/ZnIn_2_S_4_ heterostructure for efficient photocatalytic hydrogen evolution. Nanoscale.

[28] Wang W., Tadé M.O., Shao Z.P. (2018). Nitrogen-doped simple and complex oxides for photocatalysis: A review. Prog. Mater. Sci..

[29] Pan Y., Yuan X.Z., Jiang L.B., Yu H.B., Zhang J., Wang H., Guan R.P., Zeng G.M. (2018). Recent advances in synthesis, modification and photocatalytic applications of micro/nano-structured zinc indium sulfide. Chem. Eng. J..

[30] Zhu J., Liu F., Stringfollow G., Wei S.H. (2010). Strain-enhanced doping in semiconductors: Effects of dopant size and charge state. Phys. Rev. Lett..

[31] Qin J., Zhao Q., Zhao Y., Wu Y., Pan B., Wang C. (2021). Metal-free phosphorus-doped ZnIn_2_S_4_ nanosheets for enhanced photocatalytic CO_2_ reduction. J. Phys. Chem. C.

[32] Zhong L.X., Mao B.D., Liu M., Liu M.Y., Sun Y.Q., Song Y.T., Zhang Z.M., Lu T.B. (2021). Construction of hierarchical photocatalysts by growing ZnIn_2_S_4_ nanosheets on Prussian blue analogue-derived bimetallic sulfides for solar co-production of H_2_ and organic chemicals. J. Energy Chem..

[33] Chen Z., Li D., Zhang W., Shao Y., Chen T., Sun M., Fu X. (2009). Photocatalytic degradation of dyes by ZnIn_2_S_4_ microspheres under visible light irradiation. J. Phys. Chem. C.

[34] Wu Y., Yao S.K., Lv G.Z., Wang Y.W., Zhang H.J., Liao P.L., Wang Y. (2021). Construction of p-n junctions in single-unit-cell ZnIn_2_S_4_ nanosheet arrays toward promoted photoelectrochemical performance. J. Catal..

[35] Kouser S., Lingampalli S.R., Chithaiah P., Roy A., Saha S., Waghmare U.V., Rao C.N.R. (2015). Extraordinary changes in the electronic structure and properties of CdS and ZnS by anionic substitution: Cosubstitution of P and Cl in place of S. Angew. Chem. Int. Ed..

[36] Virieux H., Troedec M.L., Cros-Gagneux A., Ojo W.S., Delpech F., Nayral C., Martinez H., Chaudret B. (2012). InP/ZnS nanocrystals: Coupling NMR and XPS for fine surface and interface description. J. Am. Chem. Soc..

[37] Hu J., Lu S.H., Ma J.F., Zhu F., Komarneni S. (2022). Composite of g-C_3_N_4_/ZnIn_2_S_4_ for efficient adsorption and visible light photocatalytic reduction of Cr(VI). Environ. Sci. Pollut. Res..

[38] Zuo G.C., Ma S.S., Yin Z.Z., Chen W.Y., Wang Y.T., He H. (2023). Z-Scheme modulated charge transfer on InVO_4_@ZnIn_2_S_4_ for durable overall water splitting. Small.

[39] Chong W.K., Ng B.J., Kong X.Y., Tan L.L., Putri L.K., Chai S.P. (2023). Non-metal doping induced dual p-n charge properties in a single ZnIn_2_S_4_ crystal structure provoking charge transfer behaviors and boosting photocatalytic hydrogen generation. Appl. Catal. B Environ..

[40] Yin H.F., Cao Y., Fan T.L., Zhang M., Yao J.C., Li P.F., Chen S.M., Liu X.H. (2021). In situ synthesis of Ag_3_PO_4_/C_3_N_5_ Z-scheme heterojunctions with enhanced visible-light-responsive photocatalytic performance for antibiotics removal. Sci. Total Environ..

[41] Zhang M., Zhao X., Dong Y.Y., Hu C.Y., Xiang X.K., Zeng X.T., Jia J.H., Jin C., Ding L., Chen X.B. (2023). In-situ synthesis of 0D/1D CeO_2_/Zn_0.4_Cd_0.6_S S-scheme heterostructures for boosting photocatalytic remove of antibiotic and chromium. Ceram. Int..

[42] Lv H.J., Yin H.F., Jiao N., Yuan C.Y., Weng S.T., Zhou K.L., Dang Y.Y., Wang X.F., Lu Z., Zhang Y.Z. (2023). Efficient charge transfer and effective active sites in lead-free halide double perovskite S-scheme heterojunctions for photocatalytic H_2_ evolution. Small Methods.

[43] Wu B.G., Zhang L.P., Jiang B.J., Li Q., Tian C.G., Xie Y., Li W.Z., Fu H.G. (2021). Ultrathin porous carbon nitride bundles with an adjustable energy band structure toward simultaneous solar photocatalytic water splitting and selective phenylcarbinol oxidation. Angew. Chem. Int. Ed..

[44] Xu C.X., Kong Y.L., Zhang W.J., Yang M.D., Wang K., Chang L., Chen W., Huang G.B., Zhang J. (2022). S-scheme 2D/2D FeTiO_3_/g-C_3_N_4_ hybrid architectures as visible-light-driven photo-fenton catalysts for tetracycline hydrochloride degradation. Sep. Purif. Technol..

[45] Yin H.F., Yuan C.Y., Lv H.J., Zhang K.Y., Chen X., Zhang Y.Z. (2022). Hierarchical Ti_3_C_2_ MXene/Zn_3_In_2_S_6_ Schottky junction for efficient visible-light-driven Cr(VI) photoreduction. Ceram. Int..

[46] Pei C.Y., Li T., Zhang M., Wang J.W., Chang L., Xiong X.Q., Chen W., Huang G.B., Han D.M. (2022). Synergistic effects of interface coupling and defect sites in WO_3_/InVO_4_ architectures for highly efficient nitrogen photofixation. Sep. Purif. Technol..

[47] Yin H.F., Fan T.L., Cao Y., Li P.F., Yao X.X., Liu X.H. (2021). Construction of three-dimensional MgIn_2_S_4_ nanoflowers/two-dimensional oxygen-doped g-C_3_N_4_ nanosheets direct Z-scheme heterojunctions for efficient Cr(VI) reduction: Insight into the role of superoxide radicals. J. Hazard. Mater..

[48] Ângelo J., Magalhães P., Andrade L., Mendes A. (2016). Characterization of TiO_2_-based semiconductors for photocatalysis by electrochemical impedance spectroscopy. Appl. Surf. Sci..

[49] Yang X.X., Chen X., Cao H.L., Li C., Wang L.L., Wu Y.L., Wang C.Z., Li Y. (2020). Rational synthesis of Cu_7_Se_4_-Cu_x_Co_1-x_Se_2_ double-shell hollow nanospheres for high performance supercapacitors. J. Power Sources.

[50] Ran J., Zhang J., Yu J., Jaroniec M., Qiao S.Z. (2014). Earth-abundant cocatalysts for semiconductor-based photocatalytic water splitting. Chem. Soc. Rev..

[51] Yang J., Wang D., Han H., Li C. (2013). Roles of cocatalysts in photocatalysis and photoelectrocatalysis. ACS Chem. Res..

[52] Qi M.-Y., Conte M., Anpo M., Tang Z.-R., Xu Y.-J. (2021). Cooperative coupling of oxidative organic synthesis and hydrogen production over semiconductor-based photocatalysts. Chem. Rev..

[53] Mao Y.S., Wang P.F., Zhan S.H. (2022). Shedding light on the role of interfacial chemical bond in heterojunction photocatalysis. Nano Res..

[54] Huang W.X., Li Z.P., Wu C., Zhang H.J., Sun J., Li Q. (2022). Delaminating Ti_3_C_2_ MXene by blossom of ZnIn_2_S_4_ microflowers for noble-metal-free photocatalytic hydrogen production. J. Mater. Sci. Technol..

[55] Cai M.D., Zha X.Q., Zhuo Z.Z., Cheng Q., Wei Y.X., Sun S. (2023). Enhanced photocatalytic hydrogen production of ZnIn_2_S_4_ by using surface-engineered Ti_3_C_2_T_x_ MXene as a cocatalyst. Meterials.

[56] Zhan J.J., Gu X.Y., Zhao Y., Zhang K., Yan Y., Qi K.Z. (2023). Photocatalytic hydrogen production and tetracycline degradation using ZnIn_2_S_4_ quantum dots modified g-C_3_N_4_ composites. Nanomaterials.

[57] Hou W.Q., Chen C., Chen M., Xu Y.M. (2023). Improved activity and stability of ZnIn_2_S_4_ for H_2_ production under visible light through cerium UiO-66. Sustain. Energy Fuels.

[58] Ma Y., Xu J., Xu S.M., Liu Z.L., Liu X.Y., Li Z.Z., Shang Y., Li Q. (2023). Construction of 3D/3D heterojunction between new noble metal free ZnIn_2_S_4_ and non-inert metal NiMoO_4_ for enhanced hydrogen evolution performance under visible light. Int. J. Hydrogen Energy.

[59] Cavdar O., Baluk M., Malankowska A., Żak A., Lisowski W., Klimczuk T., Zaleska-Medynska A. (2023). Photocatalytic hydrogen evolution from glycerol-water mixture under visible light over zinc indium sulfide (ZnIn_2_S_4_) nanosheets grown on bismuth oxychloride (BiOCl) microplates. J. Coll. Inter. Sci..

[60] Wang L.L., Zhao Y.J., Zhang B., Wu G.G., Wu J., Hou H.W. (2023). Spatial separation of redox centers for boosting cooperative photocatalytic hydrogen evolution with oxidation coupling of benzylamine over Pt@UiO-66-NH_2_@ZnIn_2_S_4_. Cataly. Sci. Technol..

[61] Li Z.Z., Xu J., Liu Z.L., Liu X.Y., Xu S.M., Ma Y. (2023). 2D NiCo_2_S_4_ decorated on ZnIn_2_S_4_ formed S-scheme heterojunction for photocatalytic hydrogen production. Int. J. Hydrogen Energy.

[62] He Y.Q., Liu Y.X., Zhang Z., Wang X.Y., Li C.G., Shi Z., Feng S.H. (2023). Atomically dispersed bismuth on ZnIn_2_S_4_ Dual-Functional photocatalyst for photocatalytic hydrogen production coupled with oxidation of aromatic alcohols to aldehydes. Appl. Surf. Sci..

[63] Yu M.M., Zhang N., Zhao Y.X., Sun M., Yan T. (2023). Highly efficient visible-light photocatalytic hydrogen production using ZIF-derived Co_9_S_8_/N, S-CNTs-ZnIn_2_S_4_ composite. Chem. Phys. Lett..

[64] Lu Y., Jia X.F., Liu X.F., Zhang J.Y. (2022). W^5+^–W^5+^ pair induced LSPR of W_18_O_49_ to sensitize ZnIn_2_S_4_ for full-spectrum solar-light-driven photocatalytic hydrogen evolution. Adv. Funct. Mater..

[65] Cheng Y.S., Xing Z.Y., Yuan G.Z., Wei X.W. (2023). Integration of ReS_2_ on ZnIn_2_S_4_ for boosting the hydrogen evolution coupled with selective oxidation of biomass intermediate under visible light. Int. J. Hydrogrn Energy.

[66] Jiang Y., Li K., Wu X., Zhu M., Zhang H., Zhang K., Wang Y., Loh K.P., Shi Y., Xu Q.-H. (2021). In situ synthesis of lead-free halide perovskite Cs_2_AgBiBr_6_ supported on nitrogen-doped carbon for efficient hydrogen evolution in aqueous HBr solution. ACS Appl. Mater. Interfaces.

