# Supplementary material for: Facile Synthesis of P-Doped ZnIn2S4 with Enhanced Visible-Light-Driven Photocatalytic Hydrogen Production
Source: Molecules. 2023 Jun 2;28(11):4520. doi: 10.3390/molecules28114520 (PMC10254416; doi:10.3390/molecules28114520)
Supplement: Supplementary file 1 [file molecules-28-04520-s001.zip › molecules-2406690-supplementary.pdf]

Supporting Information

Facile synthesis of P-doped $ZnIn_2S_4$ with enhanced visible-light-driven photocatalytic hydrogen production

Xiangrui Feng, Hongji Chen, Hongfei Yin*, Chunyu Yuan, Qian Fei, Mengmeng Zheng*, Yongzheng Zhang*

School of Physics and Physical Engineering, Qufu Normal University, Qufu 273165, Shandong, China

* Corresponding author: yinhf@qfnu.edu.cn (H.F. Yin), qfzhmm@163.com (M.M. Zheng), yzzhang@qfnu.edu.cn (Y.Z. Zhang)

List of content:

Figure S1. Energy disperse spectrum elemental mapping of the pure $ZnIn_2S_4$. 2

Figure S2. XRD patterns of the as-prepared photocatalysts. 2

Figure S3. High-resolution XPS survey spectra of ZIS and P-ZIS-1.0. 3

Figure S4. BET spectra of ZIS and P-ZIS-1.0. 3

Figure S5. AQY (%) of different samples. 4

Figure S6. (a) Recycling photocatalytic H_2 generation tests over P-ZIS-1.0. (b) XRD of the fresh and used P-ZIS-1.0 samples. 4

Figure S7. (a) UV-vis DRS of the as-prepared P-ZIS; (b) The band gap value curves of the as-prepared photocatalysts obtained from UV-vis DRS spectra. 5

Table S1. Comparison of the performance of P-ZIS-1.0 with other recently reported improved materials based on ZIS. 5

Figure S8. Schematic illustration of the energy level calculation from UPS. 6

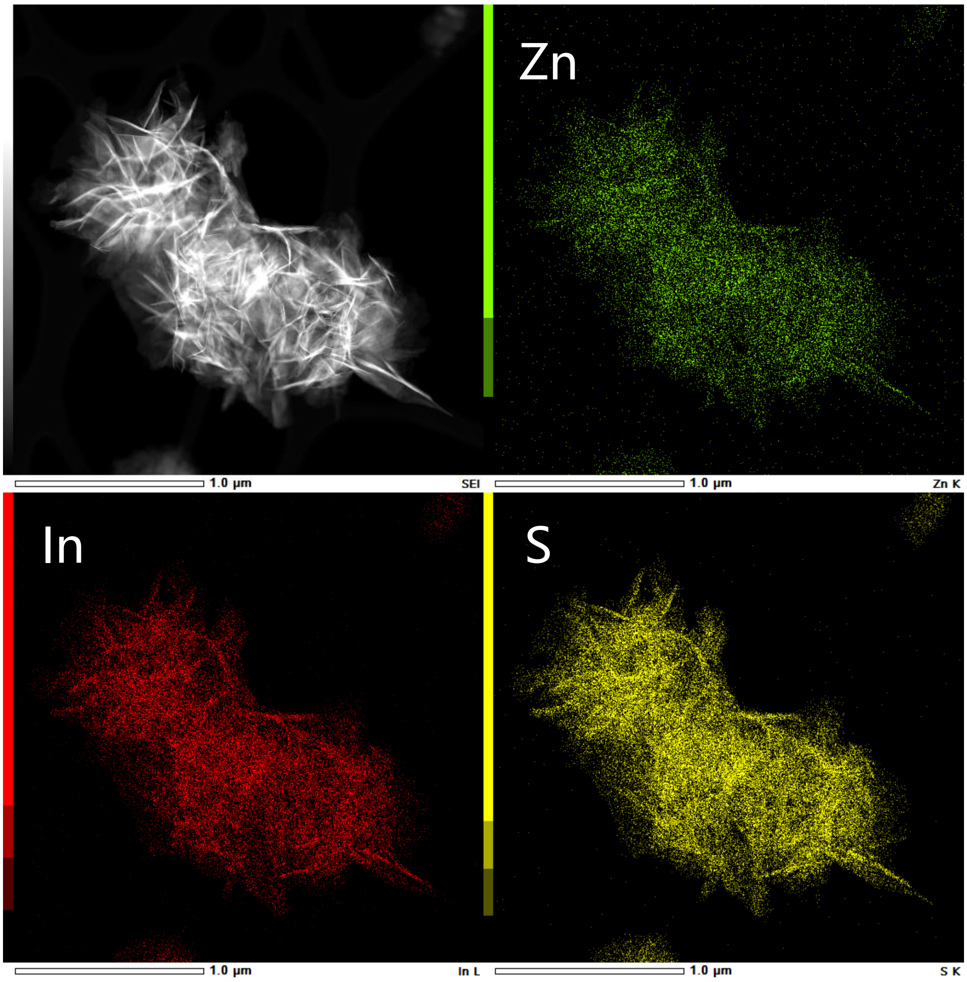

Figure S1. Energy disperse spectrum elemental mapping of the pure $ZnIn_2S_4$.

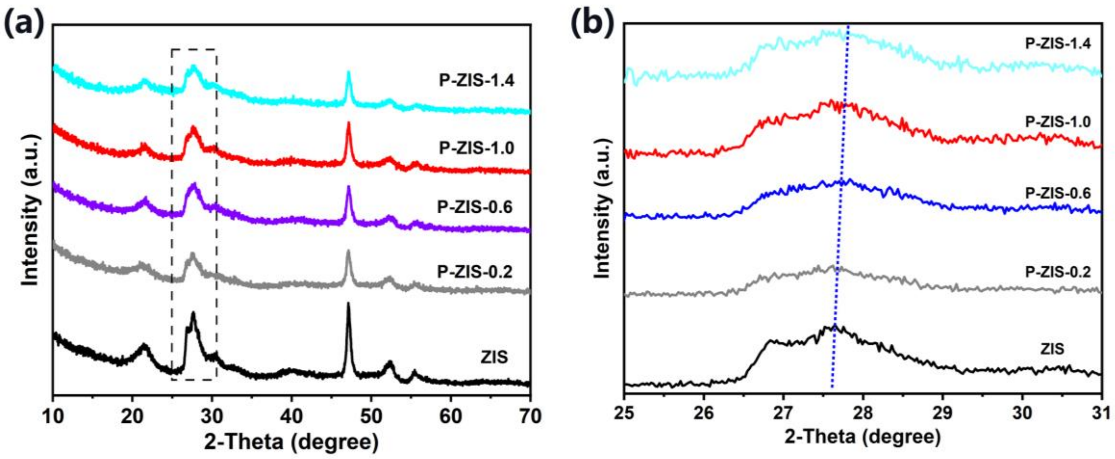

Figure S2. XRD patterns of the as-prepared photocatalysts.

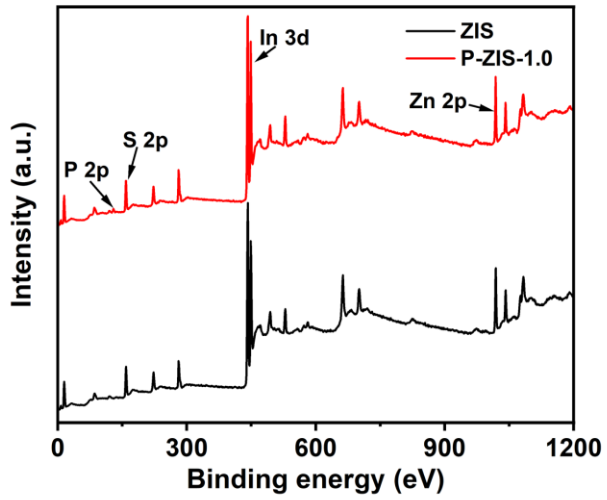

Figure S3. High-resolution XPS survey spectra of ZIS and P-ZIS-1.0.

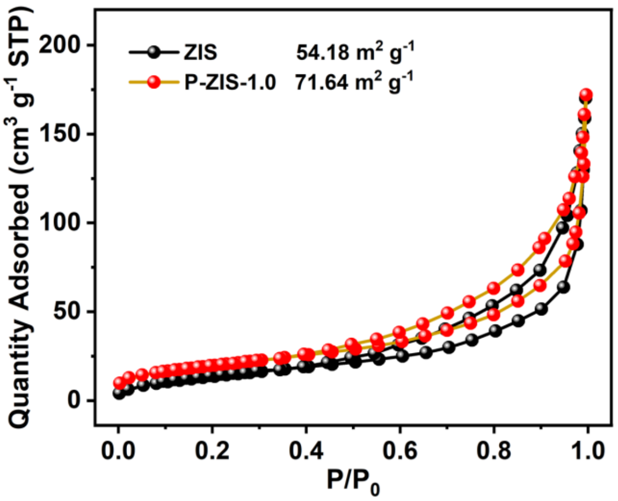

Figure S4. BET spectra of ZIS and P-ZIS-1.0.

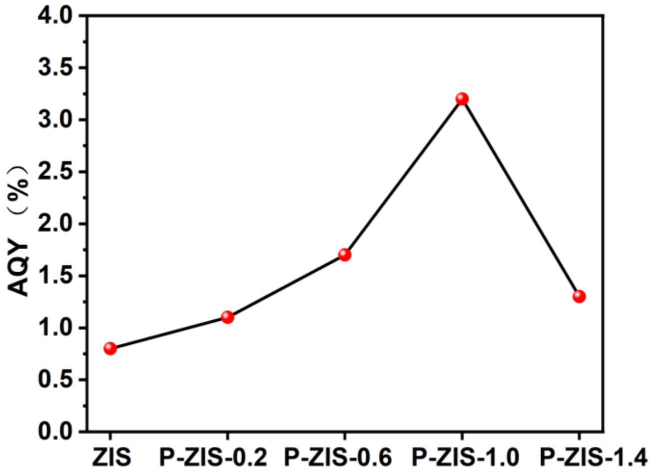

Figure S5. AQY (%) of different samples. Reaction conditions: same as photocatalytic activity test.

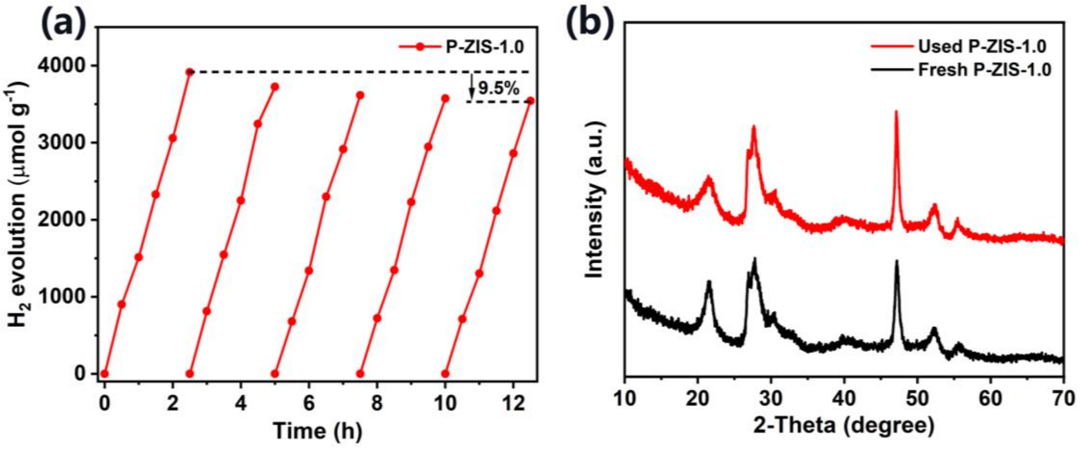

Figure S6. (a) Recycling photocatalytic H_2 generation tests over P-ZIS-1.0. (b) XRD of the fresh and used P-ZIS-1.0 samples.

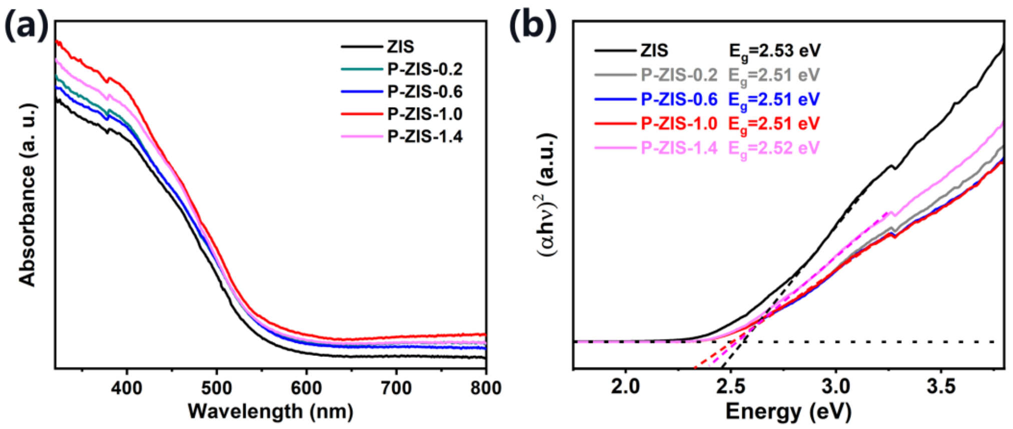

Figure S7. (a) UV-vis DRS of the as-prepared P-ZIS; (b) The band gap value curves of the as-prepared photocatalysts obtained from UV-vis DRS spectra.

Table S1. Comparison of the performance of P-ZIS-1.0 with other recently reported improved materials based on ZIS.

**Catalysts**	**Condition**	**H_2 generation (μmol g^{-1} h^{-1})**	**Year**	**Ref.**
P-ZIS-1.0	Na_2S/Na_2SO_3	1556.6	2023	This work
N-ZIS	Na_2S/Na_2SO_3	1575.7	2023	[1]
ZIS/Ti_3C_2	TEOA (10 vol%)	978.7	2022	[2]
ZIS/$Ti_3C_2O_x$	TEOA (10 vol%)	363.0	2023	[3]
20%ZIS/CN	TEOA (10 vol%)	75.2	2023	[4]
ZIS/Ce-U66	Na_2S/Na_2SO_3	500	2023	[5]
ZIS/$NiMoO_4$	TEOA (10 vol%)	140.6	2023	[6]
BiOCl@ZIS	TEOA (10 vol%)	674	2023	[7]
Pt@UiO-66-NH_2@ZIS	TEOA (10 vol%)	850	2023	[8]
$NiCo_2S_4$/ZIS	TEOA (10 vol%)	221.8	2023	[9]
Bi/ZIS	TEOA (10 vol%)	3658.8	2023	[10]
Co_9S_8/N, S-CNTs-ZIS	$K_3[Fe(CN)_6]$/ $K_4[Fe(CN)_6]$ /KCl	2409.2	2023	[11]
$W_{18}O_{49}$/ZIS	$C_3H_6O_3$	902.57	2022	[12]
ReS_2/ZIS	TEOA(10 vol%)	3092.9	2023	[13]

Calculation method for Fermi level, valence band, and conduction band [14]

UPS was used to determine the ionization energy value, the cutoff (E_{cutoff}), and the edge (E_{edge}) energy of samples. The ionization energy values (equivalent to the work function (ϕ) of metallic materials) of samples were calculated by the following equation. According to the relationship between the vacuum energy (E_{ve}) and the normal electrode potential (E_n vs NHE), the valence band energy (E_{VB} vs NHE) and conduction band energy (E_{CB} vs NHE) of samples were determined as follows:

($h\nu = \phi + E_{cutoff}$)

($E_{VB} = E_{edge} + \phi$)

($E_{CB} = E_{VB} - E_g$)

($-E_{ve} = E_n + 4.4$ eV)

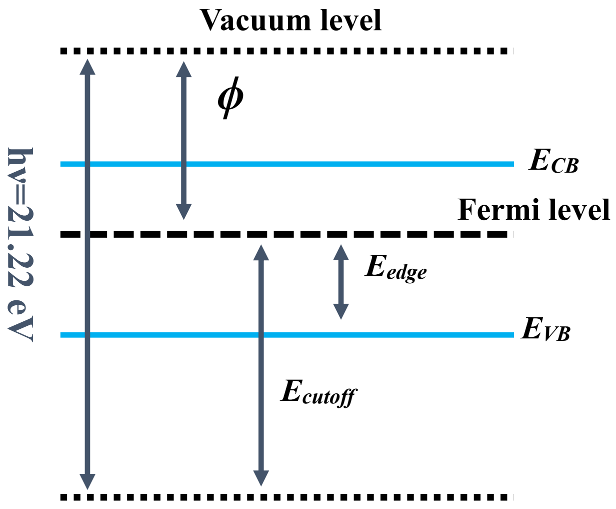

Figure S8. Schematic illustration of the energy level calculation from UPS [15].

References

[1] Chong, W.-K.; Ng, B.-J.; Kong, X. Y.; Chai, S.-P. Non-metal doping induced dual p-n charge properties in a single $ZnIn_2S_4$ crystal structure provoking charge transfer behaviors and boosting photocatalytic hydrogen generation. Appl. Catal. B: Environ. 2023, 325, 122372.

[2] Huang, W.X.; Li, Z.P.; Wu, C.; Zhang, H.J.; Sun, J.; Li, Q. Delaminating Ti_3C_2 MXene by blossom of $ZnIn_2S_4$ microflowers for noble-metal-free photocatalytic hydrogen production. J. Mater. Sci. Technol. 2022, 120, 89-98.

[3] Cai, M.D.; Zha, X.Q.; Zhuo, Z.Z.; Cheng, Q.; Wei, Y.X.; Sun, S. Enhanced photocatalytic hydrogen production of $ZnIn_2S_4$ by using surface-engineered $Ti_3C_2T_x$ MXene as a cocatalyst. Meterials 2023, 16, 2168.

[4] Zhan, J.J.; Gu, X.Y.; Zhao, Y.; Zhang, K.; Yan, Y.; Qi, K.Z. Photocatalytic hydrogen production and tetracycline degradation using $ZnIn_2S_4$ quantum dots modified g-C_3N_4 composites. Nanomaterials 2023, 13, 305.

[5] Hou, W.Q.; Chen, C.; Chen, M.; Xu, Y.M. Improved activity and stability of $ZnIn_2S_4$ for H_2 production under visible light through cerium UiO-66. Sustain. Energy Fuels 2023, 7, 1447-1453.

[6] Ma, Y.; Xu, J.; Xu, S.M.; Liu, Z.L.; Liu, X.Y.; Li, Z.Z.; Shang, Y.; Li, Q. Construction of 3D/3D heterojunction between new noble metal free $ZnIn_2S_4$ and non-inert metal $NiMoO_4$ for enhanced hydrogen evolution performance under visible light. Int. J. Hydrogen Energy 2023, 04, 11.

[7] Cavdar, O.; Baluk, M.; Malankowska. A.; Żak, Andrzej; Lisowski, W.; Klimczuk, T.; Photocatalytic hydrogen evolution from glycerol-water mixture under visible light over zinc indium sulfide ($ZnIn_2S_4$) nanosheets grown on bismuth oxychloride (BiOCl) microplates. J. Coll. Inter. Sci. 2023, 640, 578-587.

[8] Wang, L.L.; Zhao, Y.J.; Zhang, B.; Wu, G.G.; Wu, J.; Hou, H.W. Spatial separation of redox centers for boosting cooperative photocatalytic hydrogen evolution with oxidation coupling of benzylamine over Pt@UiO-66-NH_2@$ZnIn_2S_4$. Cataly. Sci. Technol. 2023, 13, 2517-2528.

[9] Li, Z.Z.; Xu, J.; Liu, Z.L.; Liu, X.Y.; Xu, S.M.; Ma, Y. 2D $NiCo_2S_4$ decorated on $ZnIn_2S_4$ formed S-scheme heterojunction for photocatalytic hydrogen production. Int. J. Hydrogen Energy 2023, 48, 3466-3477.

[10] He, Y.Q.; Liu, Y.X.; Zhang, Z.; Wang, X.Y.; Li, C.G.; Shi, Z.; Feng, S.H. Atomically dispersed

bismuth on $ZnIn_2S_4$ Dual-Functional photocatalyst for photocatalytic hydrogen production coupled with oxidation of aromatic alcohols to aldehydes. Appl. Surf. Sci. 2023, 622, 156911.

[11] Yu, M.M.; Zhang, N.; Zhao, Y.X.; Sun, M.; Yan, T. Highly efficient visible-light photocatalytic hydrogen production using ZIF-derived Co_9S_8/N, S-CNTs-$ZnIn_2S_4$ composite. Chem. Phys. Lett. 2023, 821, 140470.

[12] Lu, Y.; Jia, X.F.; Liu, X.F.; Zhang, J.Y. W^{5+}–W^{5+} pair induced LSPR of $W_{18}O_{49}$ to sensitize $ZnIn_2S_4$ for full-spectrum solar-light-driven photocatalytic hydrogen evolution. Adv. Funct. Mater. 2022, 32, 2203638.

[13] Cheng, Y.S.; Xing, Z.Y.; Yuan, G.Z.; Wei, X.W. Integration of ReS_2 on $ZnIn_2S_4$ for boosting the hydrogen evolution coupled with selective oxidation of biomass intermediate under visible light. Int. J. Hydrogrn Energy 2023, 48, 5107-5115.

[14] Jiang, Y.; Li, K.; Wu, X.; Zhu, M.; Zhang, H.; Zhang, K.; Wang, Y.; Loh, K. P.; Shi, Y.; Xu, Q.-H. In situ synthesis of lead-free halide perovskite $Cs_2AgBiBr_6$ supported on nitrogen-doped carbon for efficient hydrogen evolution in aqueous HBr solution. ACS Appl. Mater. Interfaces 2021, 13, 10037-10046.

[15] Lv, H.J.; Yin, H.F.; Jiao, N.; Yuan, C.Y.; Weng, S.T.; Zhou, K.L.; Dang, Y.Y.; Wang, X.F.; Lu, Z.; Zhang, Y.Z. Efficient charge transfer and effective active sites in lead-free halide double perovskite S-scheme heterojunctions for photocatalytic H_2 evolution. Small Methods 2023, 7, 2201365.
